# Association between β-blocker use and mortality in critically ill patients: a nested cohort study

**DOI:** 10.1186/s40360-018-0213-6

**Published:** 2018-05-16

**Authors:** Shmeylan A. Al Harbi, Khalid A. Al Sulaiman, Hani Tamim, Hasan M. Al-Dorzi, Musharaf Sadat, Yaseen Arabi

**Affiliations:** 1 Pharmaceutical Care Department, King Abdulaziz Medical City, King Saud bin Abdulaziz University for Health Sciences, King Abdullah International Medical Research Center, Riyadh, Saudi Arabia; 2 Department of Internal Medicine, American University of Beirut- Medical Center, Beirut, Lebanon; 3 Intensive Care Department, MC 1425, King Abdulaziz Medical City – National Guard Health Affairs, King Saud bin Abdulaziz University for Health Sciences, King Abdullah International Medical Research Center, PO Box 22490, Riyadh, 1426 Kingdom of Saudi Arabia

**Keywords:** β-blockers, Mortality, Sepsis, Critically ill, Intensive care, Saudi Arabia

## Abstract

**Background:**

β-blockers have several indications in critically ill patients and are commonly used. The aim of this study is to examine the relationship between the use of β-blockers in critically ill patients and mortality.

**Methods:**

This was a nested cohort study in which all medical-surgical ICU patients (*N* = 523) enrolled in a randomized clinical trial of intensive insulin therapy (ISRCTN07413772) were grouped according to β-blocker use during ICU stay. To account for the indication of β-blockers, we constructed a propensity score using selected clinically-relevant and statistically-significant variables related to β-blocker exposure and outcome. The primary endpoints were all-cause ICU and hospital mortality. Secondary endpoints were the development of severe sepsis during ICU stay, ICU and hospital length of stay, and mechanical ventilation duration. Using multivariable models, we adjusted the associations of β-blockers and these outcomes to the propensity score.

**Results:**

Of the 523 patients enrolled in the study, 89 (17.0%) received β-blockers during their ICU stay. There were no significant associations between β-blocker therapy and ICU mortality (adjusted odds ratio [aOR] 1.56, 95% confidence interval [CI] 0.83–2.9, *P* = 0.16), hospital mortality (aOR 1.09, 95% CI 0.99–1.20, *P* = 0.73), the risk of ICU-acquired severe sepsis (aOR 1.67, 95% CI 0.95–2.97, *P* = 0.08), mechanical ventilation duration (*P* = 0.17), or ICU length of stay (*P* = 0.22). However, β-blocker use was associated with increased ICU and hospital mortality among nondiabetic patients (aOR 2.93, 95% CI 1.19–7.23, and 2.43, 95% CI 1.05–5.64, respectively).

**Conclusions:**

Our study showed that β-blockers during the ICU stay had no significant association with mortality or morbidity. However, β-blocker therapy was associated with increased mortality in non-diabetic patients.

**Trial registration:**

ISRCTN07413772; (dated 13.07.2005).

## Background

β-blockers have many uses in critically ill patients. In many patients, β-blockers are used as a continuation of outpatient therapy for hypertension or cardiac indications. β-blockers are also initiated in the ICU for the management of different cardiovascular disorders and for reduction of myocardial re-infarction risk and its related mortality [1–3]. In early sepsis, β-adrenergic receptors are stimulated leading to increase in the myocardial contractility and heart rate to meet metabolic demands, but in severe cases of septic shock, cardiac depression with impaired ejection fraction and myocardial cell necrosis may occurs and is associated with increased mortality [4]. In such cases, β-blockers may reduce the deleterious effects of β-adrenergic receptor stimulation [2] and have been proposed to be used to decrease the hypermetabolic state during sepsis in general [5, 6]. However, data are limited regarding the efficacy of β-blockers in reducing mortality in critically ill patients [7].

### Objectives

This study aims to examine the relationship between use of β-blockers and mortality in medical-surgical-trauma critically ill patients.

## Methods

### Study design

This was a retrospective cohort study and post-hoc analysis of a randomized clinical trial (ISRCTN07413772) that was conducted between 2004 and 2006 and aimed to examine the effect of intensive insulin therapy on mortality in 523 medical-surgical ICU patients [8, 9]. The study was approved by King Abdullah International Medical Research Center Institutional Review Board, Riyadh, Saudi Arabia.

### Setting

This study was conducted in the adult medical-surgical ICU at King Abdulaziz Medical City, which is a tertiary-care academic referral hospital in Riyadh, Saudi Arabia. The ICU admits medical, surgical, and trauma patients, and operates as a closed unit with 24/7 onsite coverage by critical care board-certified intensivists. The nurse-to-patient ratio in the unit is approximately 1:1.2 [10]. In addition, clinical pharmacists are a part of the daily multidisciplinary rounds. Cardiac patients, including those admitted with ST elevation myocardial infraction are admitted to cardiac ICUs and are not included in this study.

### Participants

Patients were enrolled in the trial if they were ≥ 18-year-old with serum glucose level > 6.1 mmol/L (110 mg/dL) as measured by the laboratory during the first 24 h of ICU admission and had an expected ICU length of stay (LOS) > 24 h. Exclusion criteria included type I diabetes, diabetic ketoacidosis, pregnancy, “Do-Not-Resuscitate” status within 24 h of admission, terminal illness, admission to the ICU after cardiac arrest, seizures, liver transplantation and/or burn injury. All patients enrolled in the original trial were included in this analysis.

### β-blocker therapy

Data about β-blocker use in the study patients were collected from the pharmaceutical care services database and was combined with the original trial database. β-blocker therapy was either a newly prescribed medication in the ICU as per the treating teams discretion (for acute coronary syndrome or other indications) or a continuation of pre-ICU prescription. Acknowledging that different β-blockers may not have the same effect, all β-blockers were combined together because of the relatively small sample size and an expected physiological group effect. We included both oral and intravenous β-blocker use. Table 1 presents the β-blocker agents used.

**Table 1:** β-blockers used in the study

β-blocker therapy	Oral n (%)^a^	IV n (%)
Metoprolol	68 (76.4)	0
Atenolol	23 (25.8)	0
Carvedilol	1 (1.1)	0
Labetolol	1 (1.1)	12 (13.5)
Sotalol	1 (1.1)	0

^a^The sum of percentage add up to > 100% because some patients may receive another β-blocker

### Data collection

The following data were extracted from the original study: age, gender, admission category (non-operative, which included medical and trauma patients who did not require surgical interventions vs. post-operative), Acute Physiology and Chronic Health Evaluation (APACHE II) score [11], Sequential Organ Failure Assessment (SOFA) score [12] on the first day, chronic comorbidities (chronic liver disease, chronic cardiovascular disease, chronic respiratory disease, chronic renal disease and chronic immunosuppression) as defined by the APACHE II system, history of diabetes mellitus, and the presence of sepsis and severe sepsis on admission. In addition, statin and vasopressor use and serum creatinine level were documented. Estimated glomerular filtration rate (eGFR) was also calculated based on the Modification of Diet in Renal Disease equation [13].

### Outcomes

The primary outcome was all-cause ICU mortality and hospital mortality. The secondary outcomes were the development of severe sepsis [14] during the ICU stay, mechanical ventilation duration and ICU and hospital length of stay.

### Statistical analysis

There were two groups considered in this study, patients who received β-blockers during ICU admission (β-blocker group) and patients who did not (non-β-blocker group). Baseline characteristics and outcome variables were compared between the two groups. Categorical variables were presented as number and percent, whereas continuous ones were presented as mean and standard deviation. Moreover, continuous variables were compared between the two groups by the Student t-test, while categorical ones were compared by the Chi-square test.

As expected in an observational study, differences in baseline characteristics between the two study groups may exist. To adjust for these differences, a propensity score for the use of β-blockers was generated with the following variables included: age, gender, diabetes history, admission category, APACHE II score, chronic disease (respiratory disease, renal disease, chronic immunosuppression), median estimated GFR, and statin use. Multivariate logistic regression analyses was used to assess the association between β-blockers and the different outcomes considered in this study, adjusting for the generated propensity score.

Furthermore, to identify effect modification by different variables on the association between β-blockers and outcomes, we carried out subgroup analyses by the following variables: age, gender, admission category, APACHE II, history of diabetes, the presence of chronic cardiac disease, vasopressor therapy, sepsis, severe sepsis and septic shock, estimated GFR, and statin therapy. For age, APACHE II and estimated GFR, categorization was based on the median values as cut off. A *p*-value for interaction was calculated using an interaction term in the multivariate models.

Finally, the odds ratios (OR) with the 95% confidence intervals (CI) were reported for the associations.

All statistical analyses were carried out using the Statistical Analysis Software (SAS, release 9, SAS Institute, Cary, NC, 2005).

## Results

### Patient characteristics

Of the 523 patients enrolled in the study, 89 (17%) received β-blockers during their ICU stay. Table 2 presents baseline characteristics between β-blocker and non- β-blocker group. Patients who received β-blockers were older, more likely to be males, had higher APACHE II scores, had higher serum creatinine, and were more likely to be on statins. When adjusted for propensity score, all these differences became insignificant.

**Table 2:** Baseline characteristics of the β-blocker and Non-β-blocker therapy groups

Variable	β-blocker *N* = 89	Non-β-blocker *N* = 434	*P* value	PS^*^ Adjusted *P* Value
Age (years) mean ± SD	65.6 ± 14.78	49.7 ± 21.88	< 0.0001	0.37
Gender, n (%)				
Female	35 (39.3)	97 (22.4)	0.0008	0.09
Male	54 (60.7)	337 (77.7)		
Diabetes, n (%)	61 (68.5)	147 (33.9)	< 0.0001	0.31
Admission category, n (%)				
Non-operative	78 (87.6)	357 (82.3)	0.21	0.93
Post-operative	11 (12.4)	77 (17.7)		
APACHE II score, mean ± SD	24.9 ± 8.01	22.4 ± 8.09	0.008	0.48
SOFA score Day 1, mean ± SD	8.7 ± 3.65	8.8 ± 3.47	0.97	0.22
Sepsis, n (%)	18 (20.2)	104 (24.0)	0.44	0.03
Septic Shock, n (%)	23 (25.84)	102 (23.5)	0.64	0.08
Chronic Respiratory disease, n (%)	10 (11.2)	60 (13.8)	0.51	0.05
Chronic Cardiac disease n (%)	24 (27.0)	53 (12.2)	0.0003	0.16
Chronic liver disease n (%)	10 (11.2)	26 (6.0)	0.07	0.05
Chronic immunosuppression, n (%)	9 (10.1)	38 (8.8)	0.68	0.89
Chronic renal disease, n (%)	14 (15.7)	41 (9.5)	0.07	0.60
Creatinine (micromol/L), mean ± SD	194.0 ± 152.89	148.8 ± 143.10	0.01	0.52
Estimated GFR, mean ± SD	77.3 ± 62.8	54.5 ± 52.8	0.001	0.57
Vasopressor, n (%)	55 (61.8)	286 (65.9)	0.45	0.64
Statin use, n (%)	34 (38.2)	34 (7.8)	< 0 .0001	0.89

*APACHE* Acute Physiology and Chronic Health Evaluation, *SOFA* Sequential Organ Failure Assessment, *GFR* glomerular filtration rate, *PS* propensity score

*variables entered in propensity model are age, gender, diabetes history, admission category, APACHE II score, chronic disease (respiratory disease, renal disease, chronic immunosuppression), median estimated GFR, and statin use

### Outcomes

The association between β-blocker therapy and mortality using multivariate analysis adjusted for propensity score is summarized in Table 3. There was no significant association between β-blocker therapy and ICU mortality (adjusted OR 1.56, 95% CI 0.83–2.9, *P* = 0.16), hospital mortality (adjusted OR 1.09, 95% CI 0.99–1.20, *P* = 0.73), risk of ICU-acquired severe sepsis (aOR 1.67, 95% CI 0.95–2.97, *P* = 0.08), mechanical ventilation duration (*P* = 0.17), and ICU length of stay (*P* = 0.22).

**Table 3:** Outcomes of the β-blockers and non-β-blocker therapy groups

	Categorical Variables					
Variable	β-blocker *N* = 89	Non β-blocker *N* = 434	*P* value	Risk		
				aOR	95% CI	*P* value
ICU Mortality, (n%)	18 (20.2)	62 (14.3)	0.16	1.56	0.83–2.9	0.16
Hospital Mortality, (n%)	34 (38.2)	121 (27.9)	0.05	1.09	0.65, 1.83	0.73
180-day mortality, (n%)	19 (23.0)	69 (17.5)	0.25	0.73	0.38–1.38	0.33
Sepsis, (n%)	30 (33.7)	173 (39.9)	0.27	1.20	0.71–2.02	0.49
Severe Sepsis, (n%)	23 (25.8)	102 (23.5)	0.63	1.68	0.95–2.97	0.08
	Continuous Variables					
				Parameter estimate	95% CI	*P* value
Mechanical ventilation duration (days), mean ± SD	10.5 ± 13.3	8.7 ± 8.7	0.11	2.66	0.34–4.99	0.17
ICU LOS (days), mean ± SD	11.3 ± 14.0	10.0 ± 8.9	0.23	1.99	−0.43-4.41	0.22
Hospital LOS (days), mean ± SD	59.5 ± 101.9	55.0 ± 75.7	0.63	7.95	−11.68-27.58	0.34

*aOR* adjusted odds ratio, *CI* confidence interval, *ICU* intensive care unit, *LOS* length of stay

Table 4 shows the association between β-blocker therapy and all-cause hospital mortality in several subgroups of patients. The analysis showed β-blocker use was associated with increased ICU and hospital mortality among non-diabetic patients (aOR 2.93, 95% CI 1.19–7.23, and 2.43, 95% CI 1.05–5.64, respectively).

**Table 4:** Stratified analysis adjusted mortality

	180 Day Mortality				ICU Mortality				Hospital Mortality			
Variable	N	aOR	95% CI	*P* value for interaction	N	aOR	95% CI	*P* value for interaction	N	aOR	95% CI	*P* value for interaction
Age (years)												
Age < 58	230	0.57	0.10–3.2	0.58	263	1.89	0.56–6.4	0.43	263	1.21	0.41–3.5	0.27
Age > 58	247	0.83	0.43–1.62		260	1.48	0.72–3.1		260	1.11	0.62–1.99	
Gender												
Male	355	0.54	0.23–1.26	0.57	391	1.72	0.80–3.68	0.14	391	0.95	0.49–1.85	0.82
Female	122	1.45	0.52–4.1		132	1.30	0.44–3.88		132	1.45	0.62–3.39	
Diabetes												
Yes	194	0.63	0.30–1.36	0.07	208	0.93	0.40–2.17	0.01	208	0.73	0.39–1.38	0.002
No	283	1.22	0.40–3.7		315	2.93	1.19–7.23		315	2.43	1.05–5.64	
Vasopressors												
Yes	307	0.70	0.32–1.50	0.36	341	1.86	0.89–3.89	0.85	341	1.25	0.67–2.32	0.84
No	170	1.10	0.32–3.74		182	0.89	0.27–2.99		182	0.83	0.32–2.18	
APACHE II score												
< 23	236	0.85	0.22–3.26	0.32	271	2.22	0.66–7.48	0.12	271	1.12	0.40–3.13	0.23
> 23	241	0.67	0.32–1.40		252	1.23	0.59–2.56		252	0.98	0.53–1.82	
Sepsis												
Yes	117	0.83	0.24–2.95	0.37	122	1.06	0.30–3.78	0.12	122	0.92	0.32–2.63	0.06
No	360	0.71	0.33–1.51		401	1.91	0.90–4.08		401	1.26	0.70–2.37	
Severe sepsis												
Yes	115	0.52	0.11–2.41	0.71	125	0.73	0.25–2.09	0.19	125	0.71	0.25–2.01	0.72
No	362	0.80	0.39–1.62		398	2.09	092–4.73		398	1.10	0.59–2.06	
Admission category												
Non-operative	404	0.79	0.41–1.52	0.92	435	1.50	0.78–2.90	0.50	435	1.15	0.67–1.98	0.81
Post-operative	73	0.21	0.015–3.0		88	1.96	0.26–14.71		88	0.52	0.09–3.06	
Chronic Respiratory Disease												
Yes	68	1.15	0.21–6.32	0.85	70	4.89	1.18–20.30	0.11	70	7.27	1.39–37.93	0.06
No	409	0.62	0.30, 1.26		453	1.28	0.61–2.71		453	0.80	0.44–1.43	
Chronic Renal disease												
Yes	53	1.22	0.33–4.5	0.97	55	1.39	0.29–6.63	0.69	55	0.82	0.24–2.77	0.25
No	424	0.60	0.28–1.27		468	1.55	0.78–3.09		468	1.10	0.62–1.96	
Chronic Cardiac disease												
Yes	74	0.56	0.18–1.71	0.13	77	0.69	0.19–2.48	0.09	77	0.45	0.16–1.29	0.003
No	403	0.75	0.34–1.64		446	1.88	0.92–3.85		446	1.34	0.74–2.44	
Estimated GFR (mL/min)												
≤ 73	246	0.82	0.42–1.62	0.23	261	1.02	0.50–2.05	0.08	261	0.84	0.47–1.50	0.06
> 73	231	0.21	0.03–1.54		262	3.40	0.89–12.89		262	1.36	0.44–4.25	
SOFA score												
≤ 9	274	0.70	0.26–1.84	0.56	305	1.39	0.51–3.79	0.99	305	0.89	0.40–1.97	0.90
> 9	203	0.77	0.31–1.85		218	1.62	0.71–3.68		218	1.29	0.61–2.73	
ICU duration (days)												
≤ 5	231	0.74	0.28–1.92	0.70	256	1.44	0.52–4.03	0.51	256	1.07	0.46–2.47	0.31
> 5	246	0.70	0.29–1.71		267	1.60	0.71–3.58		267	1.06	0.50–2.25	
Statin Use												
Yes	66	0.92	0.30–2.79	0.86	68	0.46	0.10–2.10	0.02	68	1.005	0.35–2.92	0.19
No	411	0.65	0.28–1.51		455	2.29	1.16–4.54		455	1.40	0.76–2.57	

*aOR* adjusted odds ratio, *APACHE* Acute Physiology and Chronic Health Evaluation, *CI* confidence interval, *GFR* glomerular filtration rate, *ICU* intensive care unit, *SOFA* Sequential Organ Failure Assessment

## Discussion

Our study showed that pre-ICU continuation or initiation of β-blockers during the ICU stay in unselected critically ill patients was not associated with lower mortality or morbidity. β-blocker therapy was associated with increased mortality in non-diabetic patients.

By blocking the β-adrenergic receptors, β-blockers antagonize the effects of sympathetic nerve stimulation or circulating catecholamines [3]. Hence, they have many treatment indications and are commonly prescribed to reduce the risk of myocardial infarction and in the treatment of cardiovascular diseases, such as hypertension and congestive heart failure [1]. Additionally, they are frequently used in the general ICU setting. However, studies on β-blocker use in ICU patients varied considerably in design (retrospective or prospective cohorts, randomized controlled trials), patient population (sepsis, septic shock, postoperative), type and route of administration of β-blockers, timing of therapy (initiated pre-ICU or during ICU), target of therapy, duration of follow-up [2, 3, 7, 15–17]. As expected, these studies had different results.

Multiple studies demonstrated benefit of β-blocker use in ICU patients. Christensen et al. evaluated 8087 patients older than 45 years who were admitted to three ICUs in Northern Denmark between 1999 and 2005 and found that the pre-admission β-blocker use was associated with reduced 30-day mortality following ICU admission (OR 0.69, 95% CI 0.54–0.88 for surgical patients and 0.71, 95% CI 0.51–0.98) for medical patients [15]. Macchia et al. analyzed a database of Italian ICU patients hospitalized for sepsis and found a 28-day survival advantage in patients who were taking β-blockers at the time of admission and who subsequently developed sepsis compared with those untreated with it (OR 0.81, 95% CI 0.68–0.97; *P* = 0.025) [16]. Morelli et al. conducted the first randomized control trial in ICU patients with septic shock with a heart rate ≥ 95 beats/min and requiring high-dose norepinephrine to maintain a mean arterial pressure ≥ 65 mmHg and found that a continuous esmolol infusion, started 24 h post hemodynamic optimization and titrated to preserve the heart rate between 80 and 94 beats/min, was associated with 28-day mortality reduction and decrease in requirements of norepinephrine and fluid compared with standard care [2]. In addition, Noveanuet al. evaluated patients with acute respiratory failure who were admitted to ICU and found that oral β-blockers at admission were associated with a lower risk of both in-hospital (hazard ratio 0.33, 95% CI 0.14–0.74) *P* = 0.007) and one-year mortality (hazard ratio 0.29, 95% CI 0.16–0.51; *P* = 0.0003) [18]. In severe traumatic brain injury, β-blocker use has been associated with reduced mortality risk in multiple studies [19, 20]. A retrospective cohort study in the United States, conducted between 2000 and 2001, found that perioperative β-blocker therapy among high-risk (revised Cardiac Risk Index score ≥ 2) patients undergoing major non-cardiac surgery was associated with a reduced risk of in-hospital death (aOR for in-hospital death 0.88, 95% CI 0.80–0.98) [21].

However, other studies found worse outcomes with β-blocker use in the ICU. The POISE trial found that patients receiving metoprolol succinate 2–4 h before surgery and continued for 30 days had an increased risk of stroke and death associated with an increased incidence of hypotension, bradycardia (hazard ratio 2.17, 95% CI 1.26–3.74; *p* = 0.005) and bleeding (hazard ratio 1.33, 95% CI 1.03–1.74; *p* = 0.03) [17]. In patients older than 65 years who suffered from trauma, those without head injury, pre-injury β-blocker use was associated with increased mortality (OR 2.1, 95% CI 1.1–4.3) [22].

Our study found that β-blocker use was not associated with lower ICU mortality or hospital mortality in unselected ICU patients. Also, there was no obvious benefit in various patient subgroups, including diabetics. However, its use in non-diabetic patients was associated with increased mortality risk. β-blocker therapy among diabetic patients may have a beneficial effect. Wang et al. found that pre-operative β-blocker therapy shows a reduction in operative (0.5% vs 6.0%, *P* = 0.007) and 1 year mortality (99.1% vs 88.1%, log-rank *P* = 0.002) in patients undergoing coronary artery bypass grafting [23]. Another study by Tsujimoto et al. found a beneficial effect of β-blocker therapy in diabetic patients with history of myocardial infarction and reduced left ventricular ejection fraction (adjusted hazard ratio for all-cause mortality 0.60, 95% CI 0.37–0.98; *P* = 0.04) [24].

The findings of our study must be interpreted in the light of its strengths and weaknesses. The strengths include being a nested cohort within a randomized controlled trial with prospective data collection. On the other hand, there are some limitations in our study, including the mono-center study nature, the small sample size and the post-hoc design analysis. Although we used propensity score account for confounders, unmeasured confounders cannot be entirely be excluded. Data on the duration and doses of β-blockers prior to ICU admission and on the cardiovascular parameters were not available. Additionally, we had no long-term follow-up of patients. Also, among the questions that need answers in future studies is whether the effect of β-blocker therapy on mortality in critically ill patients is a class effect or an individual β-blocker effect.

## Conclusions

Our study showed that continuing or initiating β-blocker therapy during ICU stay was not associated with reduction in ICU or hospital mortality in unselected ICU population, but it may increase mortality in non-diabetic patients. These data confirm the need for randomized controlled trials with larger sample size to clarify the relationship between β-blocker treatment and the outcomes of critically ill patients.

## Abbreviations

aOR: Adjusted odds ratio
APACHE: Acute Physiology and Chronic Health Evaluation
DKA: Diabetic ketoacidosis
eGFR: Estimated glomerular filtration rate
ICU: Intensive care unit
LOS: Length of stay
SOFA: Sequential Organ Failure Assessment
